# The Dentist Who Came Back

**Published:** 1917-02

**Authors:** Watson W. Eldridge

**Affiliations:** New York


### THE DENTIST WHO CAME BACK.

BY WATSON W. ELDRIDGE, JR., M.D., NEW YORK.

A dentist has just left the office who will make a most excellent subject for this month's story, and whose history may not only adorn a tale, but point a moral.

He has a good strong body and a good mind. He is apparently a hustler, both physically and mentally. He works hard in the office and when freedom from the office can be gained, he plays hard at out-of-door sports. He appears to have no vices which affect his bodily welfare.

Under these conditions it might be expected that he would be a model of physical and mental health and efficiency, and indeed, for many years, and until a year or two ago, he appeared such. He then became aware of occasional periods of physical depression, without visible cause, and perceived after a time that they occurred more frequently and were more severe. He felt an unjustified fatigue. He disliked to begin work, and felt unequal to completing it. He found himself taking an occasional day away from the office when he desired neither physical nor mental activity, after his former manner; but preferred relaxation, which, however, did not appear greatly to rest him.

Exhaustive examination established the presence of an expensive auto-intoxication, of long standing, but which the body was able to throw off from time to time, and which was able to master the body only occasionally, but was gradually getting an upper hand.

Now this dentist was, in the same sense of the word, a good liver; when he had finished a hard and creditable day's work, he looked forward with pleasure to the evening meal with his family or friends. When he was particularly tired or hungry nothing so delighted his eye and appealed to his taste as a porterhouse steak about an inch and a half thick, medium broiled, with potatoes, a vegetable, a salad and dessert.

He was not what might be called an excessive eater, and if he had been occupied during all of the days in some outdoor work which could oxidize his intake, no ill effects would probably ever have attended his method of living. But his big body demanded things which the amount and kind of his work was not sufficient to oxidize, and the vital force of his digestive tract gradually failed because of the demands he unconsciously put upon it.

When the cause of his trouble was pointed out, he pooh-poohed the idea. He knew that he had always been temperate, both in the amount of his eating, and in the abstinence from the use of alcoholic liquors. Being at that time in the midst of a period of physical depression, he readily agreed to a reasonable modification of the diet, though scouting the idea that benefit could be derived from so simple and apparently unnecessary a change.

He was persuaded to reduce the amount of his meat (proteid intake) to a small portion of meat or not more than two eggs once daily, to substitute bran or whole wheat bread for white bread, to make the rest of his meal on fruit, cereals, or vegetables to his liking, and always to leave the table before he had quite satisfied his hunger.

He dropped in in passing to tell me that he felt, after his English method of expression, "quite ripping, don't you know," and had for more than two months past. I have been thinking since he went out that it is a good thing my office door opens outward, because he is so big and husky and so full now of vigor and bounce that otherwise he might not have waited to open it but might have taken it right along with him.

Only a little change, wasn't it? But it worked miracles.—*Dental Digest* for January, 1917.

